# The impact of implementing an endocarditis team in comparison to the classic heart team in a tertiary referral centre

**DOI:** 10.1186/s12872-022-02558-0

**Published:** 2022-03-18

**Authors:** F. M. A. van den Heuvel, M. Bos, G. S. C. Geuzebroek, E. H. J. G. Aarntzen, I. Maat, H. J. Dieker, M. Verkroost, L. Rodwell, J. ten Oever, R. van Crevel, J. Habets, I. J. E. Kouijzer, R. Nijveldt

**Affiliations:** 1 Department of Cardiology, Radboud University Medical Center, Geert Grooteplein Zuid 10, 6500 HB Nijmegen, the Netherlands; 2 Department of Cardiothoracic Surgery, Radboud University Medical Center, Nijmegen, The Netherlands; 3 Department of Medical Imaging, Radboud University Medical Center, Nijmegen, The Netherlands; 4 Department of Medical Microbiology, Radboud University Medical Center, Nijmegen, The Netherlands; 5 Department of Health Evidence, Section Biostatistics, Radboud University Medical Center, Nijmegen, The Netherlands; 6 Department of Internal Medicine and Radboud Center for Infectious Diseases, Radboud University Medical Center, Nijmegen, The Netherlands

**Keywords:** Infective endocarditis, Endocarditis team, Heart team, Cardiovascular imaging, Echocardiography

## Abstract

**Background:**

Infective endocarditis (IE) is a complex disease for which the European Society of Cardiology guideline recommends a dedicated multidisciplinary endocarditis team (ET) approach since 2015. It is currently unknown whether this ET approach is beneficial compared to a classic heart team approach including bedside consultation by an infectious disease specialist in Western Europe.

**Methods:**

This retrospective single centre, observational cohort study was conducted at the Radboudumc, a tertiary referral centre in the Netherlands. Consecutive patients treated for IE were included from September 2017 to September 2018 before implementation of a dedicated ET and from May 2019 to May 2020 afterwards.

**Results:**

In total, 90 IE patients (45 patients before and 45 patients after the implementation of the ET) were included. No significant differences were found in diagnostic workup, surgical treatment (surgery performed 69% vs. 71%, *p* = 0.82), time to surgery because of an urgent indication (median 4 vs. 6 days, *p* = 0.82), in-hospital complications (53% vs. 67%, *p* = 0.20), and 6-month mortality (11% vs. 13%, *p* = 0.75) between IE patients treated before and after the implementation of the ET.

**Conclusion:**

Formalization of the recommended multidisciplinary endocarditis team might not significantly improve the complication rate nor the short term outcome.

**Supplementary Information:**

The online version contains supplementary material available at 10.1186/s12872-022-02558-0.

## Background

Infective endocarditis (IE) is a disease with a high in-hospital mortality, morbidity, and complication rate [1–4]. Despite treatment improvements over the last decades, severe complications are still common [5]. The diagnosis of IE is challenging and is based on imaging, microbiological results and clinical criteria. For diagnostic classification, the European Society of Cardiology (ESC) 2015 modified diagnostic criteria are used [5]. The main treatment of IE consists of intravenous antibiotics [5, 6] and cardiac surgery is indicated in approximately 50% of patients [6–8]. Because of the challenging diagnosis and the complexity of the disease, a multidisciplinary approach is needed. In 2015, ESC guidelines were updated for the management of IE and a dedicated Endocarditis Team (ET) was introduced [5]. This multidisciplinary team consists of several specialties, including at least a cardiologist, cardiac surgeon, specialist in infectious diseases, microbiologist, anesthesiologist, and nuclear medicine physician. Several studies suggest that this multidisciplinary approach does not only result in a decrease in mortality, but also reduces complication rate and improves treatment defined as time to surgery [9–11]. The ESC guidelines for the management of IE also includes recommendations regarding timing of surgery classified as emergency, urgent, or elective [5]. Since the release of the ESC guidelines in 2015, many centers have implemented the recommendations, with the Radboudumc implementing them in 2019. The objective of this study was to assess the implementation of a dedicated ET on ESC guideline adherence and outcome. In addition, we compared our diagnostic work up and outcome data with the European observational research programme European endocarditis (EURO-ENDO) registry [8], a large multicentre prospective cohort study. The implementation of our dedicated ET was evaluated on diagnostic work-up, treatment, mortality rate, and complication rate compared to our previous heart team approach.

## Methods

### Patient population

This single-centre, observational cohort study was conducted at the Radboud university medical centre (Radboudumc), a tertiary referral centre for infectious diseases and cardiothoracic surgery. All consecutively hospitalized patients treated for IE during one year before (15 September 2017 to 15 September 2018) and during one year after (15 May 2019 to 15 May 2020) the implementation of the ET were included. In the arbitrary 7 months period between, no patients were enrolled as this was the start-up phase of the multidisciplinary ET. All patients included in the second period were evaluated at least once by the ET to discuss diagnostics and treatment strategy. Patients below the age of 18 years and patients with a cardiac implantable electronic device (CIED) infection were excluded. The regional institutional ethics committee approved this study and the requirement to obtain informed consent was waived (nr. 2019–5224).

### Data collection

Patient data, including demographics, medical history, diagnostics, microbiological results, antibiotic treatment, time to surgery, complications occurring during admission at the Radboudumc, and mortality 6 months after IE diagnosis were collected. All data were retrieved electronically from the electronic medical records (EMR) and reports of diagnostic studies. The IE patients in the period before the ET were identified by manually searching the database from the outpatient parenteral antimicrobial therapy (OPAT) team and cardiothoracic surgery. This was complemented by, an extensive search in our electronic medical records using CTCUE software (Amsterdam, the Netherlands). In this search all patients which were hospitalised during this 1 year perioded with the word endocarditis in the EMR were screened for eligibility. In the period after the implementation of the dedicated ET, IE patients were identified from the ET meeting lists in the EMR. Patients were only included once in the study. Six months mortality rate was retrieved form the population registry for all patients. Additional data on surgery, readmission, and relapse after discharge were gathered from the EMR of the Radboudumc.

### Outcome measures

The primary objective of this study was the comparison of patients before and after the implementation of the ET on baseline characteristics, diagnostic work-up, antimicrobial and surgical treatment, in hospital complications, and 6-month mortality. Second, we compared our diagnostic work-up and outcome data with the European observational research programme European endocarditis (EURO-ENDO) registry [8].

### Definitions

IE was diagnosed according to the ESC 2015 modified diagnostic criteria [5]. The first day of IE was defined as the day that imaging was positive for IE. Imaging modalities used were transthoracic echocardiography (TTE), transoesophageal echocardiography (TEE), [18F]-fluorodeoxyglucose positron emission tomography-computed tomography ([^18^F]FDG-PET/CT), or (cardiac) computed tomography angiography (CTa). Imaging was performed either at the Radboudumc or referring hospitals. Blood cultures were available in all patients. The following complications occurring during hospitalization at the Radboudumc were registered: embolic events, heart failure, cardiogenic shock, intracranial haemorrhage, renal failure, and death. Embolic events were defined as clinical or subclinical metastatic infections shown on any imaging modality. Heart failure was defined as clinical signs or symptoms consistent with heart failure. Cardiogenic shock was defined as a state of impaired end-organ perfusion, due to a reduced cardiac output. Renal failure was defined as a new glomerular filtration rate < 30 mL/min/1.73 m^2^ or the need of renal dialysis. Time to surgery was defined as the period between IE diagnosis and the actual operation date. Indications for surgery were according to ESC guideline [5] and were divided into the indication “heart failure”, “uncontrolled infection” and “prevention of embolism” according to guideline. If patients did not met one of those three indications for surgery they were classified as “other”. If more than one surgical indication was present according to the ESC guideline [5] in one patient, the timing category (emergent: < 24 h, urgent: < 7 days or elective: after 1–2 weeks of antibiotic therapy) was assessed by using the most emergent indication. In case of a new complication which changed the indication and/or timing for surgery according to the ESC guideline, time to surgery was defined as the period between complication and the operation date. Relapse was defined as a new episode of IE with the same microorganism after the end of antibiotic treatment. Re-infection was defined as a new episode of IE with another microorganism after the end of antibiotic treatment. All patients received antibiotic treatment according to institutional guidelines that are in line with the national guideline (www.swabid.nl).

### Endocarditis and classic heart team

In the period before the implementation of the dedicated ET, all IE patients were discussed in the local classic heart team. The heart team discusses patients every working day or ad hoc in critically ill patients. The heart team consists of an interventional cardiologist, an imaging cardiologist and a cardiothoracic surgeon. In the period after implementation of the ET, all hospitalized IE patients at the Radboudumc and referred IE patients were discussed once a week in the ET. Our ET consists of cardiologists, cardiac surgeons, infectious disease (ID) specialists, clinical microbiologists, and nuclear medicine physicians. An anaesthesiologist was consulted in case of an operation indication. In case of a probable indication for surgery, patients were also discussed in the heart team where the final decision for cardiac surgery was made. In all patients, before and after implementation of the ET, bedside consultation by an infectious disease specialist was performed.

### Statistical analysis

Continuous variables are presented as median and interquartile range (IQR). Categorical variables are presented as counts and percentages. Median values were compared between groups using the Mann–Whitney *U* test. Categorical values were compared between groups using a χ^2^ test. A power analysis or a minimal sample size calculation was not performed as this study was designed as an observational cohort study in which two time periods of one year are compared. Propensity score matching was not used because we would not expect a difference in patient population. The survival across both groups was compared using the Kaplan–Meier survival analysis. A one sample T-test was used to compare our groups with the values derived from the EURO-ENDO registry. A *P *value < 0.05 was considered statistically significant. All statistical analyses were performed using SPSS statistics (IBM SPSS Statistics 25, Armonk, NY, USA).

## Results

### Study population

In this observational study, 90 patients treated for IE were included, of which 45 patients were included in the period before and 45 patients in the period after implementation of the ET. The median age of all patients was 65 years (IQR 53–71) and 73% were male. Other baseline characteristics are shown in Table 1. Twenty-six patients (29%) were diagnosed at the Radboudumc and 64 patients (71%) were diagnosed in a referring hospital. According to the ESC 2015 modified diagnostic criteria [5], 76% of patients had definite, 22% possible, and 2% rejected IE. The affected valve was a native valve in 53% and a prosthetic valve in 48% (Table 1). Of all 32 IE patients in the ET period who underwent surgery, 14 patients (44%) underwent surgery first and were discussed afterwards in the ET, 15 patients (47%) were first discussed in the ET and underwent surgery afterwards, and the remaining 3 patients (9%) underwent surgery on the same day of the ET.

**Table 1:** Baseline characteristics of all IE patients

	All IE patients n = 90 (%)	Before ET patients n = 45 (%)	After ET patients n = 45 (%)	*P* value before ET patients versus after ET patients
Sex, male	66 (73)	30 (67)	36 (80)	0.15
Age (years), median (IQR)	65 (53–71)	63 (51–70)	66 (54–73)	0.31
*Comorbidities*				
Cardiac history				
Moderate- to severe valvular disease	9 (10)	6 (13)	3 (7)	0.29
Prosthetic valve	43 (48)	23 (51)	20 (44)	0.33
CABG	10 (11)	4 (9)	6 (13)	0.50
Bentall	12 (13)	8 (18)	4 (9)	0.22
Congenital heart disease	17 (19)	10 (22)	7 (16)	0.42
Bicuspid aortic valve	11 (12)	7 (16)	4 (9)	0.33
Cardiac electronic device	9 (10)	5 (11)	4 (9)	0.73
Diabetes mellitus	20 (22)	8 (18)	12 (27)	0.31
Active cancer	5 (6)	4 (9)	1 (2)	0.17
Chronic renal failure	4 (4)	3 (7)	1 (2)	0.31
Chronic obstructive pulmonary disease (COPD)	9 (10)	7 (16)	2 (4)	0.08
Immunosuppressive therapy	7 (8)	4 (9)	3 (7)	0.69
*Echocardiography*				
TTE performed	82 (91)	41 (91)	41 (91)	1.0
TTE result				
Positive	23 (28)	12 (29)	11 (27)	0.81
Undetermined	47 (57)	24 (59)	23 (56)	0.82
Negative	12 (15)	5 (12)	7 (17)	0.53
TEE performed	62 (69)	30 (67)	32 (71)	0.65
TEE result				
Positive	44 (71)	22 (73)	22 (67)	0.69
Undetermined	10 (16)	4 (13)	6 (19)	0.56
Negative	8 (13)	4 (13)	4 (13)	0.92
Vegetation seen on TTE or TEE	56 (62)	27 (60)	29 (64)	0.66
Intracardiac complication seen on TTE or TEE	22 (24)	11 (24)	11 (24)	1.0
Valve perforation	8 (9)	3 (7)	5 (11)	0.46
Aortic root abscess	8 (9)	5 (11)	3 (7)	0.46
Prosthetic valve dehiscence or paravalvular regurgitation	2 (2)	1 (2)	1 (2)	1.0
Fistula	1 (1)	0	1 (2)	0.32
*Other diagnostics used*				
FDG PET-CT	46 (51)	22 (49)	24 (53)	0.67
CTangio aorta	16 (18)	9 (20)	7 (16)	0.58
Cardiac CT	7 (8)	4 (9)	3 (7)	0.69
CT Thorax and/or abdomen	18 (20)	9 (20)	9 (20)	1.0
CT brain	15 (17)	6 (13)	9 (20)	0.40
MRI brain	14 (16)	6 (13)	8 (18)	0.56
*Type of valve infected*				
Native valve	48 (53)	22 (49)	26 (58)	0.40
Prosthetic valve	43 (48)	23 (51)	20 (44)	0.53
Biological valve	24 (56)	12 (52)	12 (60)	0.61
Mechanical valve	17 (40)	10 (44)	7 (35)	0.57
TAVI	2 (5)	1 (4)	1 (5)	0.92
Lead/patch involved^a^	2 (2)	0	2 (4)	0.15
Aortic valve involved	64 (71)	32 (71)	32 (71)	1.0
Mitral valve involved	29 (32)	17 (38)	12 (27)	0.26
Tricuspid valve involved	2 (2)	1 (2)	1 (2)	1.0
Pulmonary valve involved	5 (6)	3 (7)	2 (4)	0.65
*Microbiology*				
Duration of antibiotic treatment (days)	43 (42–53)	42 (42–55)	43 (37–49)	0.67
Blood cultures positive	76 (84)	36 (80)	40 (89)	0.25
Pathogen in blood cultures				
*Staphylococcus aureus*	17 (22)	8 (22)	9 (23)	0.98
Coagulase-negative *Staphylococci*	4 (5)	3 (8)	1 (3)	0.26
*Streptococci*	40 (53)	19 (53)	21 (53)	0.98
*Enterococci*	6 (8)	3 (8)	3 (8)	0.73
HACEK group	2 (3)	0	2 (5)	0.17
Other	7 (9)	3 (8)	4 (10)	0.80

Values are in median and interquartile range, or n (%)

CABG, coronary artery bypass graft; CT, computed tomography; FDG PET/CT, [18F]-fluorodeoxyglucose positron emission tomography-computed tomography; GFR, glomerular filtration rate; SCAR, supracoronary aorta ascendens replacement; TAVI, transcatheter aortic valve implantation; TEE, transoesophageal echocardiography; TTE, transthoracic echocardiography

^a^Patients with a valve IE primarily with cardiovascular implantable electronic device infection

### Diagnostic work-up, treatment, and complications in all IE patients

Echocardiography was performed in 99% of all patients. TTE was performed in 91% and TEE in 69% of all patients. On echocardiography vegetations were seen in 62% and intracardiac complications in 24% (Table 1). [^18^F]FDG-PET/CT was used in 51% of all patients of which 57% was positive for IE. [^18^F]FDG-PET/CT showed bone or joint foci in 23%, metastatic pulmonary foci in 11%, and splenic foci in 7%. Cardiac surgery was performed in 63 patients (70%) (Table 2). Of these patients, 39 patients (62%) had native valve IE (NVE) and 24 patients (38%) had prosthetic valve IE (PVE). The most common timing category for surgery was ‘urgent’ (89%) and the median time from urgent indication to surgery in all patients was 5 days (IQR 2–8). Of all patients who underwent surgery, heart valve cultures were taken in 92% of which 35% cultures were positive. Complications during hospitalization occurred in 60%, with embolic event as the most frequent complication (31%) (Table 2). The median duration of antibiotic treatment was 43 days (IQR 42–53).

**Table 2:** Treatment and outcome of valve IE patients

	All IE patients n = 90 (%)	Before ET patients n = 45 (%)	After ET patients n = 45 (%)	*P* value before ET patients versus after ET patients
*Cardiac surgery performed*	63 (70)	31 (69)	32 (71)	0.82
Indication for surgery				
Heart failure	25 (40)	12 (39)	13 (41)	0.88
Uncontrolled infection	22 (35)	11 (36)	11 (34)	0.93
Prevention of embolic event	14 (22)	7 (23)	7 (22)	0.95
Other	2 (3)	1 (3)	1 (3)	0.98
Type of surgery performed				
AVR	26 (41)	11 (36)	15 (47)	0.36
MVR	13 (21)	7 (23)	6 (19)	0.71
AVR and MVR	4 (6)	2 (7)	2 (6)	0.97
Bentall / biological composite graft	15 (24)	10 (32)	5 (16)	0.12
Other	5 (8)	1 (3)	4 (9)	0.17
*Timing of surgery*				
Indication				
Emergent	0	0	0	–
Urgent	56 (89)	28 (90)	28 (88)	0.72
Urgent/elective	1 (2)	0	1 (3)	0.32
Elective	6 (10)	3 (10)	3 (9)	0.97
*Urgent surgery*				
Time from urgent indication to surgery, days	5 (2–8)	4 (2–8)	6 (2–8)	0.82
Surgery within 24 h after indication	9 (16)	4 (14)	5 (18)	0.72
Surgery within 1 to 3 days after indication	11 (20)	6 (21)	5 (18)	0.74
Surgery within 4 to 7 days after indication	19 (40)	10 (36)	9 (32)	0.59
Surgery 7 or more days after indication	17 (30)	8 (29)	9 (32)	0.77
*In hospital complications*	54 (60)	24 (53)	30 (67)	0.20
Embolic event	28 (31)	12 (27)	16 (36)	0.81
Acute heart failure	14 (16)	5 (11)	9 (20)	0.45
Cardiac shock	4 (4)	1 (2)	3 (7)	0.42
Intracranial hemorrhage	5 (6)	0	5 (11)	0.04
Renal failure	21 (23)	12 (27)	9 (20)	0.13
Deceased during hospitalization	5 (6)	2 (4)	3 (7)	0.65
*Follow-up*				
Readmission after initial discharge	17 (19)	7 (16)	10 (22)	0.42
Relapse or re-infection	5 (6)	2 (4)	3 (7)	0.65
Thoracic surgery after initial discharge	8 (9)	4 (9)	4 (9)	1.0
Deceased within 6 months after diagnosis	11 (12)	5 (11)	6 (13)	0.75
*Discharge*				
Duration of hospital admission (days)	17 (11–34)	17 (10–34)	19 (11–34)	0.97

Values are in median and interquartile range, or n (%)

AVR, aortic valve replacement; MVR, mitral valve replacement

^a^Patients who underwent cardiac surgery without valve replacement, e.g. drainage of cardiac abscess or valve repair

### Follow up

**Fig. 1:**
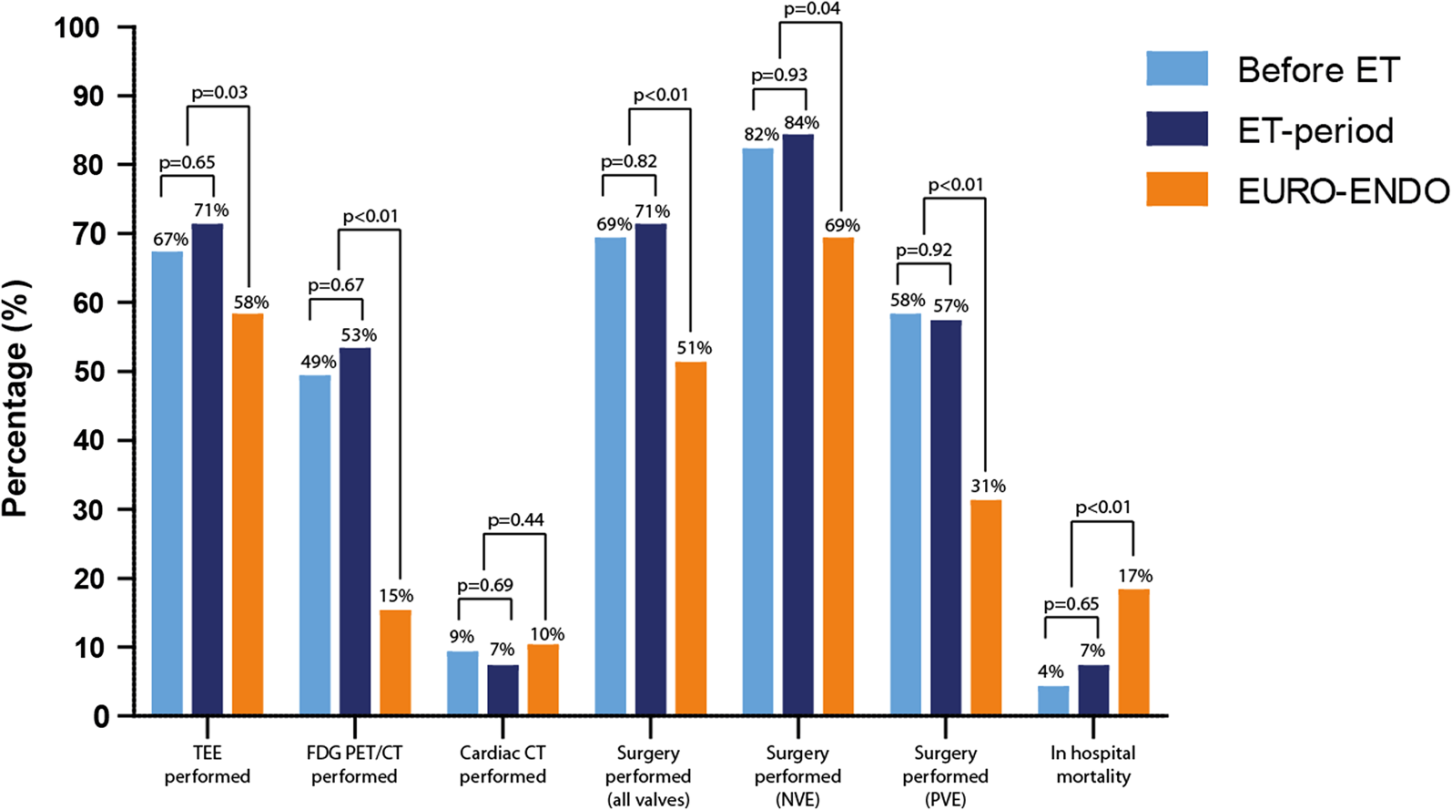
Comparing valve IE patients between our cohort and EURO-ENDO study. CT; computed tomography, EURO-ENDO: European observational research programme European endocarditis registry; FDG PET/CT: [18F]-fluorodeoxyglucose positron emission tomography-computed tomography; IE; infective endocarditis; NVE: native valve endocarditis; TEE: transoesophageal echocardiogram; PVE: prosthetic valve endocarditis. Values are in %

Of all 90 patients, 11 patients (12%) deceased within 6 months after diagnosis; 5 patients (6%) deceased during hospitalisation, and 6 patients (7%) died after initial discharge (Table 2). Mortality rates are also shown in a Kaplan–Meier curve (Fig. 1). In total, 17 patients (19%) were readmitted to the hospital after initial discharge, due to IE or a complication. Two patients (2%) had a relapse and 3 patients (3%) a re-infection. Eight patients (9%) underwent thoracic surgery after initial discharge.

### Comparison before and after implementation ET

Comparing the groups before and after the implementation of the ET, there were no differences in baseline characteristics, diagnostic work-up, antimicrobial and surgical treatment, in hospital complications, and six-month mortality (Tables 1, 2, 3). Analysing time to surgery showed that in patients with an urgent operation indication, surgery was performed in 71% within < 7 days, compliant with the ESC guidelines, whereas after formalization of the dedicated ET this was 68% (Table 2).

**Table 3:** Complications in patients with an indication to undergo urgent surgery before and after implication of the Endocarditis Team

	Before ET patients n = 28 (%)	After ET patients n = 28 (%)	*P* value before ET patients versus after ET patients
Complications	19 (68)	20 (71)	0.78
Embolic event	10 (53)	11 (55)	0.88
Acute heart failure	3 (16)	8 (40)	0.10
Cardiac shock	0	3 (15)	0.08
Intracranial hemorrhage	0	3 (15)	0.08
Renal failure	10 (53)	5 (25)	0.08
Deceased during hospitalization	1 (4)	2 (7)	0.55
Deceased within 6 months after diagnosis	4 (14)	4 (14)	1.0

### Subgroup analysis

Time to surgery and in hospital complication rate were not different between all patients diagnosed in the Radboudumc compared to those diagnosed in a referring hospital (Additional file 1: Data). Surgery was performed more often in all patients diagnosed in a referring hospital (86%) compared to all patients diagnosed in the Radboudumc (31%) (*p ≤ *0.01). Four patients (16%) deceased during hospitalization in the group diagnosed in the Radboudumc, where one patient (2%) deceased in the group diagnosed in a referring hospital. Another subgroup analysis between all patients who underwent surgery within and after 7 days of urgent indication showed no significant differences in hospitalization duration and complication rates (Additional file 1: Data). In the group of patients in whom surgery was postponed for more than 7 days, 4 patients (24%) had contra-indications for surgery (temporary contraindication for surgery (n = 2), expert opinion to postpone surgery (n = 2)). By excluding these patients, the compliance to the ESC guideline according to urgent surgery in both groups of patients was 75%.

### EURO-ENDO study

**Fig. 2:**
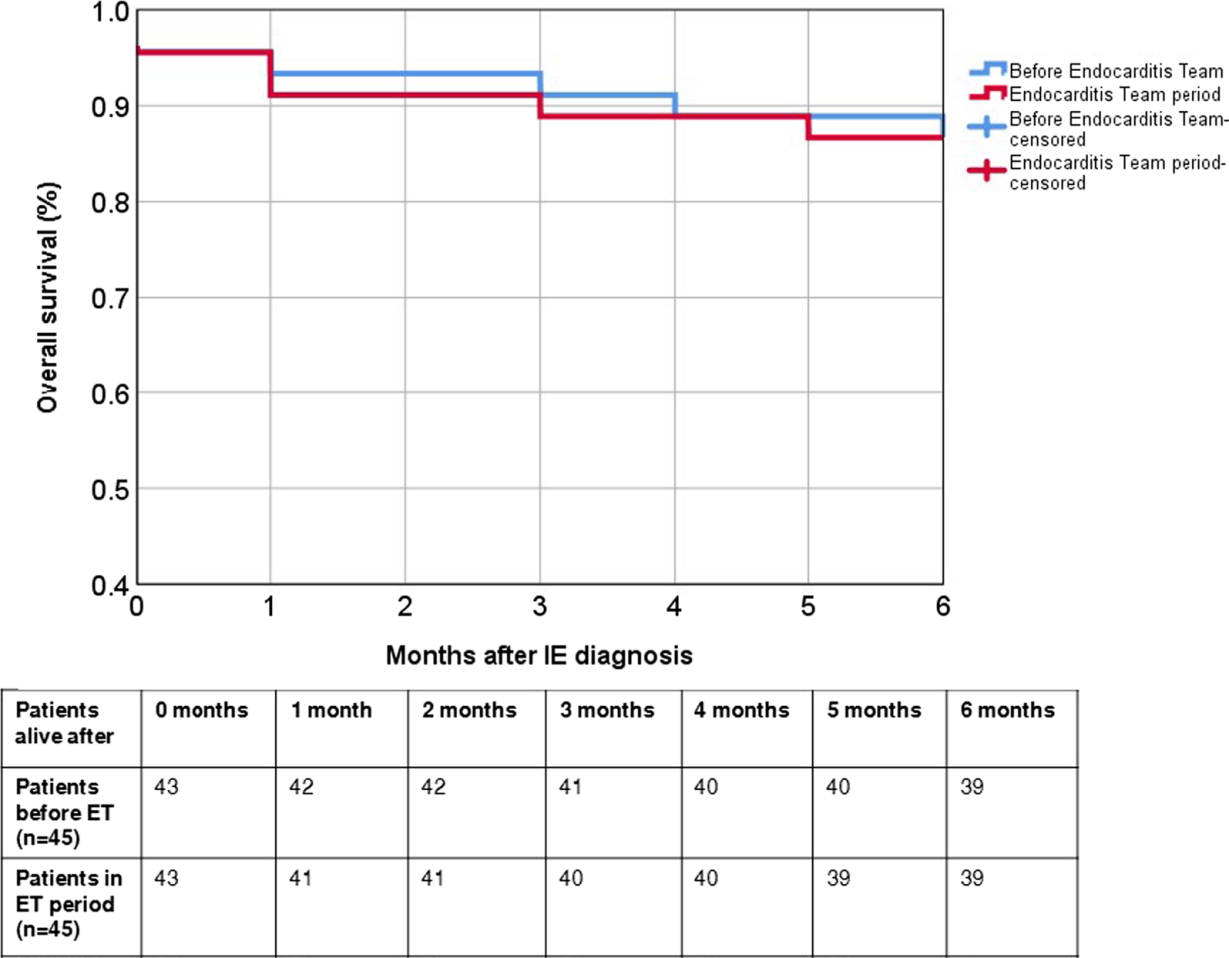
Survival among groups before and after Endocarditis Team. Kaplan–Meier curve comparing survival before and after implementation of the endocarditis team. *P *value at 6 months: 0.75

Our results were compared to the cohort of native and prosthetic valve IE patients of the EURO-ENDO registry [8]. Age, gender, comorbidities, and microbiology results were numerically comparable. Chronic renal failure occurred more often in the EURO-ENDO population (17%) than in our population (4%). The number of native valve endocarditis was slightly different, with 65% in EURO-ENDO and 53% in our cohort, respectively (Fig. 2). Compared to the EURO-ENDO registry the use of [^18^F]FDG-PET/CT is more frequent in our centre (15% vs 51%, *p ≤ *0.01). Some outcomes could not be compared, such as time to surgery and complications, as definitions differed. Our results could not be compared to specific countries in the EURO-ENDO registry as the EURO-ENDO registry only provides results per country for the whole group of IE including device infections.

## Discussion

In this observational cohort study we evaluated the implementation of the guideline recommended dedicated ET on diagnostic work-up, treatment, complications, and mortality of IE in a tertiary referral centre compared to a classic heart team approach including bedside consultation by an infectious disease specialist. Our single center experience demonstrates that the implementation of a multidisciplinary ET might not significantly improve the complication rate nor the short term outcome. Further prospective, multicenter studies which also evaluate long term outcome are needed in this regard. Comparing our results to the EURO-ENDO registry, we reported a higher [^18^F]FDG-PET/CT usage and a lower in-hospital mortality.

In contradiction to our results, several other studies have shown that the implementation of a multidisciplinary approach is beneficial. First, Botelho-Nevers et al. [9] demonstrated in 2009 a reduction in 1-year mortality of 10% (from 19 to 8%) after implementation of a multidisciplinary protocol. However, this reduction might also have been influenced by different antimicrobial regimen, different duration of treatment, different surgical indications, and influenced by exclusion of rare microorganisms causing IE. Second, Chirillo et al. showed a decrease in 3-years mortality (from 34 to 16%) after implementation of a surgical protocol and a multidisciplinary approach in native valve IE patients [12]. Third, Kaura et al. demonstrated a decrease in time to surgery (from 8 to 5 days) and an increase in 12-month survival in medically managed patients only (43–67%) after implementation of an ET [10]. Fourth, Ruch et al. showed a reduction in time to surgery (16–10 days) after setup of an ET [11]. The first possible explanation for the difference between our results and the aforementioned studies could be the fact that those studies only included definite IE defined by the modified Duke criteria. As we did not select our study population based on diagnostic criteria, but included all patients treated for IE, as possible and rejected cases were also included in our study. A second explanation could be a difference in the set-up of the ET. Chirillo et al. evaluated all patients in the ET within 12 h after diagnosis. Kaura et al. discussed new referrals directly, existing cases once a week in the ET, and reviewed all cases in ward rounds twice a week. We have discussed IE patients only once a week in the ET, and critically ill patients were discussed immediately in the daily classic heart team. As a result of this policy, decisions on surgery and surgery timing had already been taken in the heart team (44% underwent surgery before discussion in the ET). Another more plausible explanation for our results may be that our diagnostic approach before the implementation of the ET, was already multidisciplinary and of high quality, as shown by a low in-hospital mortality rate in the period before the ET. Even before the start of the ET, there was daily availability of highly qualified ID specialists [13] and advanced cardiovascular imaging was also already used. In our opinion, this has caused the limited potential impact and expected value of the multidisciplinary ET, since the multidisciplinary collaboration was only differently and more formally organized.

Comparing our results to the EURO-ENDO registry [8], we reported a higher [^18^F]FDG-PET/CT usage (51% compared to 15%), and a lower in-hospital mortality (6% compared to 17%). An explanation for the increase in [^18^F]FDG-PET/CT -usage, is that our centre is highly experienced in using [^18^F]FDG-PET/CT in infectious diseases. A possible explanation for the decrease in in-hospital mortality could be that the difference in patient population. The EURO-ENDO registry included only definite IE (84%) or possible IE which were considered and treated as IE (16%) based the ESC 2015 diagnostic criteria. Our study included all patients treated for IE (76% of patients had definite, 22% possible, and 2% rejected IE). Another possible explanation for the decrease in in-hospital mortality could be that the higher use of [^18^F]FDG-PET/CT identifies more metastatic infections, which potentially leads to treatment modification and therefore to a lower mortality [14].

Subgroup analysis showed that surgery was performed more often in patients diagnosed in a referring hospital compared to patients diagnosed in the Radboudumc. This difference is explained by the fact that our hospital is a referral centre for cardiothoracic surgery and thereby the primary referral reason is an indication for surgery.

Although we found no differences in patient outcomes, we still recommend a dedicated ET based approach for multiple reasons. First, because of the complexity of the disease which warrants a multidisciplinary strategy and leads to a more efficient decision-making regarding treatment. Second, in our experience a second opinion regarding the diagnostic work-up and treatment strategy is warranted because of the potentially high complication and mortality rate. Third, the ET leads to improved interdisciplinary and regional collaboration. Last, the ET is useful to reject the diagnosis of IE and therefore to avoid unnecessary diagnostics and treatment. However, based on our findings, a classic heart team (i.e. cardiothoracic surgeon, imaging and interventional cardiologist) including bedside consultation by an ID specialist can be considered as a safe alternative for the ET, provided that there is a good collaboration and clear communication.

## Conclusions

In conclusion, in our daily practice formalization of a multidisciplinary ET approach might not improve diagnostic work-up, treatment, complication rate, mortality, and short-term outcome in IE patients compared with a classic heart team approach including bedside consultation by an ID specialist.

### Study limitations

Our study has some limitations. First, patients in the period before the ET were included using a retrospective search in our EMR. Therefore, some patients might have been missed. A second limitation is its single-centre observational design and relatively small sample size. Third, selection bias might have occurred, as we only included patients admitted to the Radboudumc, a tertiary referring hospital. Fourth, we have only analysed outcome results in the first year of the ET. After more years of multidisciplinary team work and experience, the quality and short term outcome of the ET might be further improved. Last, CIED infections were not included because of the relatively small number of patient with CIED infections on one hand, and incompleteness of the data of CIED infections in the period before the ET on the other, limiting the interpretability of the results.

## Supplementary Information

**Additional file 1.** Subgroup analysis.

## Data Availability

The data underlying this article will be shared on reasonable request to the corresponding author.

## Abbreviations

[^18^F]FDG-PET/CT: [18F]-fluorodeoxyglucose positron emission tomography-computed tomography
AVR: Aortic valve replacement
CABG: Coronary artery bypass graft
CIED: Cardiac implantable electronic device
COPD: Chronic obstructive pulmonary disease
CTa: Computed tomography angiography
EMR: Electronic medical records
ESC: European Society of Cardiology
ET: Endocarditis team
EURO-ENDO: European observational research programme European endocarditis
GFR: Glomerular filtration rate
ID: Infectious disease
IE: Infective endocarditis
IQR: Interquartile range
MVR: Mitral valve replacement
NVE: Native valve IE
OPAT: Outpatient parenteral antimicrobial therapy
PVE: Prosthetic valve IE
TAVI: Transcatheter aortic valve implantation
TEE: Transoesophageal echocardiograghy
TTE: Transthoracic echocardiography
SCAR: Supracoronary aorta ascendens replacement
