# Supplementary material for: The impact of implementing an endocarditis team in comparison to the classic heart team in a tertiary referral centre
Source: BMC Cardiovasc Disord. 2022 Mar 18;22:114. doi: 10.1186/s12872-022-02558-0 (PMC8931961; doi:10.1186/s12872-022-02558-0)
Supplement: Supplementary file 1 — Additional file 1. Subgroup analysis. [file 12872_2022_2558_MOESM1_ESM.docx]

Supplemental data

## Subgroup analyses

|  | **Patients diagnosed in Radboudumc vs in referring hospital** | **All urgent patients who underwent surgery within 7 days and ≥7 days after indication** | **Definite IE patients before vs after** **implementation of the ET** |
| --- | --- | --- | --- |

|  | **Radboudumc n=26 (%)** | **Referring hospital n=64 (%)** | ***P*-values Radboudumc vs referring hospital** | **Within 7 days  n=30 (%)** | **After 7 days or more  n=26 (%)** | ***P*-values before vs after 7 days** | **Definite before ET n=30 (%)** | **Definite after ET  n=38 (%)** | | ***P*-values before vs after ET** |
| --- | --- | --- | --- | --- | --- | --- | --- | --- | --- | --- |
| Time to surgery | 5 (0-10) | 6 (2-14) | 0.33 | 4 (2-6) | 8 (1-17) | 0.04 | 7 (2-18) | 6 (3-12) | | 0.38 |
| Cardiac surgery performed | 8 (31) | 55 (86) | <0.01 | 30 (100) | 26 (100) | >0.99 | 22 (73) | 27 (71) | | 0.84 |
|  |  |  |  |  |  |  |  | |  |  |
| **Complications** | 12 (46) | 42 (66) | 0.09 | 22 (73) | 17 (65) | 0.52 | 20 (67) | 27 (71) | | 0.70 |
| Embolic event | 6 (23) | 22 (34) | 0.88 | 11 (50) | 10 (59) | 0.58 | 12 (40) | 14 (37) | | 0.58 |
| Acute heart failure | 2 (8) | 12 (19) | 0.41 | 6 (27) | 5 (29) | 0.88 | 4 (13) | 8 (21) | | 0.45 |
| Cardiac shock | 2 (8) | 2 (3) | 0.17 | 1 (5) | 2 (12) | 0.40 | 1 (3.) | 3 (8) | | 0.46 |
| Intracranial hemorrhage | 1 (4) | 4 (6) | 0.90 | 2 (9) | 1 (6) | 0.71 | 0 | 5 (13) | | 0.04 |
| Renal failure | 4 (15) | 17 (27) | 0.65 | 9 (41) | 6 (35) | 0.72 | 9 (30) | 9 (24) | | 0.42 |
| Deceased during hospitalization | 4 (15) | 1 (2) | 0.01 | 2 (7) | 1 (4) | 0.64 | 2 (7) | 3 (8) | | 0.85 |
| Deceased within 6 months after diagnosis | 4 (15) | 7 (11) | 0.56 | 3 (10) | 5 (19) | 0.33 | 4 (13) | 5 (13) | | 0.98 |

AVR: aortic valve replacement; MVR: mitral valve replacement. Values are in median and interquartile range, or n (%).
